# Late Presentation of Dyskeratosis Congenita: Germline Predisposition to Adult-Onset Secondary Acute Myeloid Leukemia

**DOI:** 10.3390/hematolrep14040042

**Published:** 2022-10-02

**Authors:** Harry Ramos, Mai Mostafa Aly, Suresh Kumar Balasubramanian

**Affiliations:** 1 School of Medicine, Wayne State University, Detroit, MI 48201, USA; 2 Clinical Hematology Unit, Department of Internal Medicine, Assiut University, Assiut 71515, Egypt; 3 Department of Oncology, School of Medicine, Wayne State University, Detroit, MI 48201, USA

**Keywords:** dyskeratosis congenita, AML, telomere, *FLT3*-TKD, del 7

## Abstract

Classic dyskeratosis congenita is a hereditary disease where the majority of patients present with bone marrow failure and mucocutaneous changes: mainly skin pigmentation, nail dystrophy, oral premalignant leukoplakia, in addition to increased risk for malignancies. A 63-year-old man with a long history of untreated chronic pulmonary disease, a smoker in the past, presented initially with pancytopenia and a clinical diagnosis of myelodysplastic syndrome with excess blasts returned a month later with leukocytosis (WBC 215.9 × 10^6^/μL) and diagnosed with acute myeloid leukemia (AML) with deletion of chromosome 7 and *FLT3*-TKD mutation. The patient’s mother and sister died in their 6th decade from rapidly progressing fulminant pulmonary fibrosis. He had abnormal skin pigmentation and oral leukoplakia on presentation. He was induced with 7 + 3 chemotherapy and started on midostaurin but experienced prolonged cytopenias, complicated by hypoxic acute on chronic respiratory failure requiring intubation and mechanical ventilation. D + 28 and D + 36 bone marrow examination showed trilineage hypoplasia but no blasts, though the D + 28 bone marrow biopsy revealed one metaphase with del (7) that was cleared on D + 35. The constellation of clinical features and strong family history along with del 7 and *FLT3*-TKD AML with preceding MDS highly suggests a germline predisposition state dyskeratosis congenita. Germline predispositions are often underrecognized as delayed onset conditions leading to AML and may have treatment and preventative implications especially genetic counseling for blood-related family members.

## 1. Introduction

**Figure 1:**
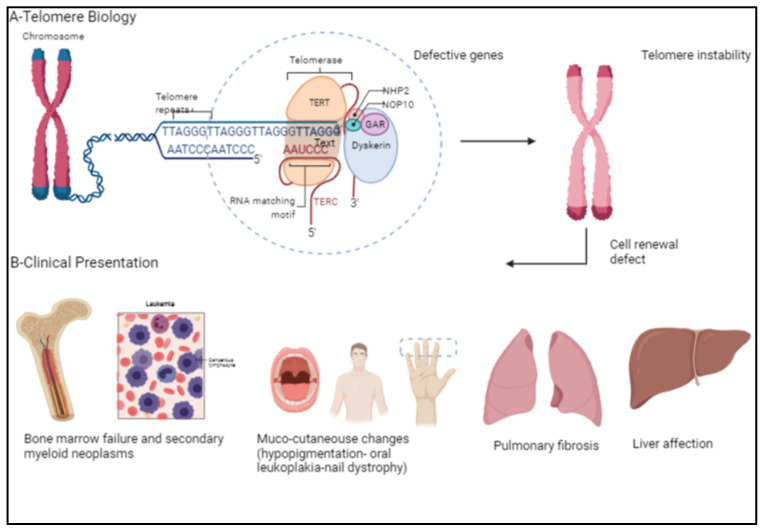
(**A**) Demonstrates telomere biology and the various genes involved in telomere elongation. (**B**) Telomere instability causes classical clinical presentations in patients with dyskeratosis congenita, namely bone marrow failure, secondary myeloid neoplasms, mucocutaneous changes, pulmonary fibrosis, and liver disease. [Created with BioRender.com] (BioRender, Toronto, ON, Canada).

Dyskeratosis congenita (DC) presents with a constellation of bone marrow failure (BMF) and mucocutaneous manifestations, including skin pigmentation, nail dystrophy, and oral premalignant leukoplakia, in addition to increased risk for malignancies (Figure 1). DC is a disease with a heterogenous inheritance pattern depending on the mutation(s) within the chromosomal telomeres. DC can be inherited as autosomal dominant (AD) if mutations are in the *TERC* or *TERT* genes, as X-linked recessive (XLR) if the *DKC1* gene is mutated, or as autosomal recessive (AR) where various other mutated genes have been implicated but have not been entirely determined [1,2,3,4]. Pulmonary fibrosis (PF) is frequent and seen in 65% of DC patients, characterized by patchy, basal, and peripheral reticular opacity, ground glass changes, and honeycombing on high-resolution computerized tomography [5,6]. The liver is frequently affected, presenting as cirrhosis, portal hypertension, or steatosis [7,8,9].

DC is characterized by telomere shortening, either a disruption in the telomere’s inherent length or a set of gene mutations coding proteins involved in telomere maintenance. Human telomere (TTAGGG) caps the terminal ends of chromosomes that protect the DNA from degradation by endonucleases or the harsh oxidative environment stress during normal cellular processes. It is widely known that telomere length correlates with the replicative capacity such that cells cease to divide—reach cellular senescence—or undergo apoptosis when telomeres reach a critically short length. Understanding this biological behavior of telomeres provides insight into the disease processes that highly involve the proliferative nature of hematopoietic and dermatological systems that considers DC as a premature aging state [10].

In this case report, we present a possible case of delayed presentation of DC. This patient was diagnosed with del 7 and *FLT3*-TKD mutated AML with myelodysplastic changes. He also had oral leukoplakia, abnormal skin pigmentation, liver pathology, and a strong family history of fatal PF. The patient suffered a long history of pulmonary disease and was further complicated by respiratory failure requiring mechanical ventilation, likely from the effect of chemotherapy and post-therapy neutropenic infections in an underlying diseased lung.

## 2. Case Report

**Figure 2:**
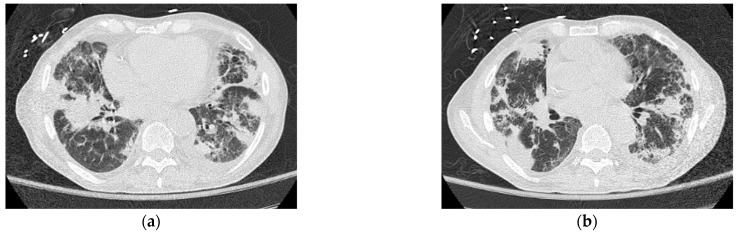
(**a**,**b**) Computerized tomography of the thorax demonstrating patient’s irregular pulmonary infiltrates, with peripheral cystic changes (honeycombing) and traction bronchiectasis.

A 63-year-old Caucasian man with an untreated lung disease presented with generalized fatigue and not feeling well. His initial labs revealed cytopenias [Hgb: 10.2 g/dL, WBC: 8.9 K/CUMM, and platelets: 37 K/uL] which on initial evaluation showed dysplastic myeloid lineage cells in the peripheral blood. Failing to follow further workup, he presented to a local emergency room 4 weeks later with leukocytosis [215.5 × 10^6^ per microliter] and pancytopenia [Hgb: 7.1 g/dL, ANC: 4300/uL, and Platelets: 8 K/uL]. He had circulating blasts [65% blasts], and bone marrow examination revealed AML with MDS-related changes and deletion 7 [20/20 metaphases] on karyotyping, and next-generation sequencing identified an *FLT3*-TKD mutation. The patient’s blast crisis was treated with leukapheresis at an outside facility before being induced with 7 + 3 chemotherapy. The course was complicated by worsening respiratory failure requiring intubation and mechanical ventilation. Chest imaging revealed pulmonary infiltrates (Figure 2a,b), which were not resolving, and the patient had persistent supplemental oxygen requirements even after several days since extubation. He had unusually prolonged pancytopenia, and a D + 28 bone marrow biopsy revealed bone marrow hypoplasia with decreased trilineage hematopoiesis but no evidence of blasts on flow cytometry. However, he was noted to have one metaphase positive for del (7q). He was treated with antibiotics, antivirals, and appropriate mold prophylaxis and then transferred to our facility on D + 34 for lack of bone marrow recovery. D + 38 bone marrow examination revealed similar findings to the earlier biopsy but no del (7q) evidence. Table 1 demonstrates the patient’s lab values showing cytopenia despite treatment indicating the patient’s progressing bone marrow failure.

**Figure 3:**
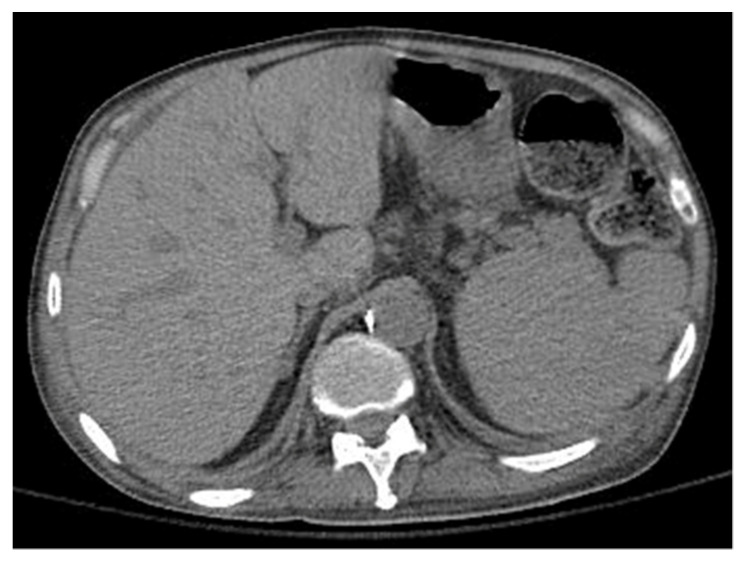
Computerized tomography of the liver shows steatosis with diffuse hepatocellular change.

Further probing revealed that the patient’s history of pulmonary disorder was never treated nor investigated. Physical examination revealed abnormal skin hypopigmentation and oral leukoplakia. The patient’s radiographic studies showed scoliosis with advanced degenerative spondylosis and steatosis of the liver with diffuse hepatocellular changes despite having no history of alcoholism (Figure 3). Of note, family history was significant for the patient’s mother and sister dying of rapidly progressing fulminant PF in their 50 s.

Throughout the hospitalization, the patient remained cytopenic, spiked fever, and eventually succumbed to respiratory failure and complications of prolonged neutropenia. The patient could not be screened for telomere mutations though his clinical conundrum was strongly suggested DC. The patient’s biological children were counseled and referred to a telomere clinic for appropriate genetic counseling and screening.

## 3. Differential Diagnosis

The constellation of BM suppression, PF, and chronic history of mucocutaneous abnormalities such as skin hypopigmentation and oral leukoplakia led to considering a germline predisposition condition preceding the patient’s AML with MDS-related changes in the differential: namely, Fanconi anemia (FA), Schwachman–Diamond Syndrome (SDS), and DC. Although FA is commonly diagnosed during childhood, it has been reported to penetrate later in adulthood and is associated with an increased risk of developing AML. FA, characterized by skin pigmentation, head and neck abnormalities, and BM suppression with increased risk of AML, lacks the pulmonary fibrosis seen in DC. SDS has varied presentations, like the clinical conundrum of our patient. However, the classic abnormality of pancreatic insufficiency and malabsorption of SDS were absent in our patient. Although genetic testing can confirm the diagnosis of DC, the symptoms, physical findings, progression of the disease, and convincing family history are strongly suggestive of the late presentation of this fatal illness for our patient.

## 4. Discussion

DC’s inheritance varies, but all patterns of inheritance affect the biological processes that maintain the integrity of chromosomal telomeres. Mutations in telomerase have been implicated in DC. Other genes complex with telomerase pathways, such as *DKC1, TERC, TINF2, NOLA2,* and *NOLA3,* can also be mutated in DC. *TERC* is commonly mutated in patients with AD and AR forms of DC, aplastic anemia, myelodysplasia syndrome (MDS), and paroxysmal nocturnal hemoglobinuria. *TERC* mutations suggest that the abnormal telomerase activity levels and telomeres length affect DC’s varied clinical phenotype presentation [11,12].

Cytopenia is the first sign of BMF, and DC patients usually risk developing MDS and AML [13]. Chromosome 7 abnormalities are the most commonly reported cytogenetic abnormalities in BMFs. Interestingly, 12% of DC patients had monosomy 7, and 3% developed MDS [14]. Telomere length regulation may also be linked to the pathogenesis of −7/del (7q). Telomere attrition precedes monosomy 7, and the accumulation of short telomeres gives rise to aneuploidy leading to leukemogenesis [9,14]. Our patient presented with del 7 and cytopenia, which likely preceded his leukemia.

The varying progression of the disease further complexes the diverse symptomology. The median age of DC diagnosis is 15 years; however, a subset of patients, particularly with the AD inheritance, present at a later age, a case of disease anticipation [4,11]. Familial PF is known to have an AD inheritance pattern with incomplete penetrance manifesting at an early age, in contrast to sporadic idiopathic PF. The strong family history of immediate blood-related family members succumbing to rapid onset fulminant PF in their 6th decade raises the strong possibility that our patient likely had an AD form of DC with haploinsufficiency; most likely may have harbored *TERC* or *TERT* mutations as pulmonary fibrosis is expected in this type of DC [10].

If diagnosed earlier, differentiation between DC and other BMF syndromes is crucial as DC does not respond well to immunosuppressive therapy [15]. However, most patients respond to androgens with improved hematological parameters, including red blood cells and platelets [15,16]. However, allogeneic hematopoietic stem cell transplantation (Allo-HSCT) remains the only curative treatment for DC, though myeloablative conditioning regimens can be highly toxic [17]. Graft versus host disease, graft failure, infection, veno-occlusive disease, and pulmonary complications can negatively affect Allo-HSCT outcomes of DC. Reduced-intensity conditioning (RIC) with a fludarabine-based regimen and matched related donors are associated with favorable outcomes [17,18]. Allo-HSCT can cure BMF and reduce the possibility of secondary MDS/AML but does not impact the mucocutaneous, lung, and liver manifestations. Our patient suffered from prolonged pancytopenia post-chemotherapy, reasonably justifying a reduced intensity AML induction treatment but may need prospective clinical studies to substantiate that approach.

This case illustrates an atypical late presentation of DC. Despite the inability to assess the telomere length and presence of DC mutations, the clinical course strongly suggests DC, which implies that a good medical and family history and a physical examination in adjunct to the appropriate genetic testing are necessary to diagnose patients with a possible germline AML predisposition state.

## 5. Conclusions

In summary, our patient with a strong positive family history of fulminant fatal PF and a personal history of prolonged untreated pulmonary disease, mucocutaneous changes, liver steatosis, and deletion 7/*FLT3*-TKD mutated AML with MDS-related changes is a syndrome highly suggestive of late presentation of adult type DC. While most adult AML cases are often considered sporadic and treated with conventional doses of chemotherapy, a clinical picture that raises a strong suspicion of germline predisposition should trigger an appropriate evaluation, even in an older patient. Such an approach may reveal the submerged portion of the iceberg, which may have treatment and preventative implications for patients and their immediate family members.

## Figures and Tables

**Table 1:** Hematological laboratory values of the patient (BM bx = bone marrow biopsy; PB = peripheral blood).

	D-42 (BM bx)	D0 (PB)	D + 27 (PB)	D + 28 (BM bx)	D + 36 (BM bx)
WBC (K/CUMM)	8.7	215	0.2	0.1	0.4
RBC (M/CUMM)	2.73	1.4	2.89	2.2	2.65
Hemoglobin (gm/dL)	8.1	4.3	8.3	6.3	7.7
Hematocrit (%)	23.9	13	24	18.8	23.6
MCV (FL)	105	98	83	85.5	89.1
RDW (%)	15.5	16	14.8	14.9	17
Platelets (K/CUMM)	37	1	2	9	3
MPV (FL)	11.5	11	10.2	11.1	
Reticulocyte (/CUMM)	3600			3800	
Abs lymphocyte count					0.2
Abs monocyte count	0.1				0
Abs neutrophil count	0.4	0.1			0.4
Blasts (%)	18	65			

## Data Availability

The data used to support the findings of this study are included in the article.
